# Accuracy of random-forest-based imputation of missing data in the presence of non-normality, non-linearity, and interaction

**DOI:** 10.1186/s12874-020-01080-1

**Published:** 2020-07-25

**Authors:** Shangzhi Hong, Henry S. Lynn

**Affiliations:** Department of Biostatistics, Key Laboratory on Public Health Safety of the Ministry of Education, School of Public Health, Fudan University, Shanghai, China

**Keywords:** Missing data imputation, Imputation accuracy, Random forest

## Abstract

**Background:**

Missing data are common in statistical analyses, and imputation methods based on random forests (RF) are becoming popular for handling missing data especially in biomedical research. Unlike standard imputation approaches, RF-based imputation methods do not assume normality or require specification of parametric models. However, it is still inconclusive how they perform for non-normally distributed data or when there are non-linear relationships or interactions.

**Methods:**

To examine the effects of these three factors, a variety of datasets were simulated with outcome-dependent missing at random (MAR) covariates, and the performances of the RF-based imputation methods missForest and CALIBERrfimpute were evaluated in comparison with predictive mean matching (PMM).

**Results:**

Both missForest and CALIBERrfimpute have high predictive accuracy but missForest can produce severely biased regression coefficient estimates and downward biased confidence interval coverages, especially for highly skewed variables in nonlinear models. CALIBERrfimpute typically outperforms missForest when estimating regression coefficients, although its biases are still substantial and can be worse than PMM for logistic regression relationships with interaction.

**Conclusions:**

RF-based imputation, in particular missForest, should not be indiscriminately recommended as a panacea for imputing missing data, especially when data are highly skewed and/or outcome-dependent MAR. A correct analysis requires a careful critique of the missing data mechanism and the inter-relationships between the variables in the data.

## Background

Missing data are common in clinical and public health studies, and imputation methods based on machine learning algorithms, especially those based on random forest (RF) are gaining acceptance [1]. In the original article, the RF-based missing data imputation R package missForest is described as an imputation algorithm designed for mixed continuous and/or categorical data in the presence of complex interactions and non-linearity without requiring to specify the distributions of the variables [2]. Another R package CALIBERrfimpute allowed for multiple imputation by sampling from conditional distributions constructed using RF [3].

Several studies have reported that missForest performed favorably compared to standard imputation methods, and missForest has been used as a benchmark for non-parametric imputation methods [4, 5]. In a comparison study done by Waljee et al. [6], missForest was found to consistently produce the lowest imputation error compared with other imputation methods, including *k*-nearest neighbors (*k*-NN) imputation and “mice” [7], when data were missing completely at random (MCAR). Tang and Ishwaran also recommended missForest when variables have high inter-correlations [5]. Yet Shah et al. [3] reported that missForest produced substantially biased estimates for variables missing at random (MAR) and poor coverage of confidence intervals compared with CALIBERrfimpute. Solaro et al. [8] demonstrated that the relative performance of missForest varied with the MCAR data patterns and did not show a clear advantage. Overall, the imputation accuracy and applicability of missForest is still unclear. Moreover, the differences between CALIBERrfimpute and missForest imputation on statistical analyses warrant further investigation.

This study evaluates the imputation accuracy of missForest and CALIBERrfimpute in the presence of non-normally distributed variables, interaction, or non-linearity when data are MAR [9]. The examination was done through a series of simulation experiments and a case study based on clinical data for patients with hepatocellular carcinoma (HCC).

## Methods

### Imputation methods

#### MissForest

The missForest algorithm can be summarized as follows:

(1) Initialization. For a variable containing missing values, the missing values will be replaced with its mean (for continuous variables) or its most frequent class (for categorical variables).

(2) Imputation. The imputation process is done sequentially for the variables in the data in ascending (or descending, if appointed) order of missing observations for each variable. The variable under imputation is used as the response for building the RF model. The observations in the dataset are divided into two parts according to whether the variable is observed or missing in the original dataset. The observed observations are used as the training set, and the missing observations are used as the prediction set. The missing part of the variable under imputation is replaced by predictions from RF models [10, 11].

(3) Stop. When all the variables with missing data have been imputed then one imputation iteration is completed. The imputation process is iterated until the relative sum of squared differences (or proportion of falsely classified entries for categorical variables) between the current and the previous imputation results increases, and missForest outputs the previous imputation as the final result [2]. A maximum number of iterations user setting with a default value of 10 is also installed in the process to limit the computational time to a reasonable level.

#### MICE-based imputation

Both CALIBERrfimpute and predictive mean matching (PMM) operate under the framework of multivariate imputation using chained equations (MICE), with different MICE imputation methods differing in the process they use to impute missing values. The typical process of MICE-based imputation can be summarized as:

(1) Initialization. For a variable containing missing values, the missing values will be replaced with random samples from observed values of that variable.

(2) Imputation. The imputation process is done sequentially for the variables according to their original order in the dataset. The variable under imputation is used as the response for model building. The observations in the dataset are divided into two parts according to whether the variable is observed or missing in the original dataset. The observed observations are used as the training set, and the missing observations are used as the prediction set. The missing part of the variable under imputation is replaced by the imputed values generated from the user-specified MICE-based imputation method.

(3) Stop. When all the variables with missing data have been imputed then one imputation iteration is completed. The imputation process is iterated until the maximum number of iterations (default value of 5) is reached, and the final result is the last imputation.

The PMM method is a semi-parametric imputation method recommended as the default imputation method by the “mice” R package [7] to serve as a “baseline” method for comparison. For each variable, PMM calculates the predicted regression values for its non-missing and missing observations. It then fills in a missing value by randomly selecting one from the non-missing observations whose predicted values are closest to the predicted value for the missing observation. The purpose of the regression in PMM is to construct a metric for matching observations with missing values to similar observations with observed values that can be used for imputation.

CALIBERrfimpute was included to investigate the influences of imputation using the conditional distribution of the RF prediction errors. To form conditional distributions using RF, for each variable CALIBERrfimpute assumed normality for the RF prediction errors and used the out-of-bag mean square error as the estimator of their variance. Variables with missing values are imputed by random draws from independent normal distributions centered on the conditional means predicted by RF. It should be noted that to focus on the comparison with the prediction of imputed values in missForest, the additional bootstrapping of observed data in CALIBERrfimpute to account for sampling variation in multiple imputation was not used.

In this study, five iterations were performed for each imputation within the MICE framework as small iteration numbers were recommended [7]. Also, for the RF-based imputation methods, the number of trees built was set to ten as suggested by Shah et al. [3] for less biased results.

### Simulation study

Data simulation and statistical analyses were carried out using R (R Core Team, Vienna, Austria) [12], and four sequential stages were involved:

1. Data generation: complete datasets were simulated based on each of the predefined scenarios for each of the distributions.
2. Amputation: the complete datasets were made incomplete based on specified rules to generate missing data values.
3. Imputation: the missing values of the simulated incomplete datasets were filled-in by different imputation methods, resulting in imputed complete datasets.
4. Analysis: statistical analyses were performed on both the original complete datasets and the corresponding imputed datasets, and comparisons of the different imputation methods were made.

#### Data generation

**Fig. 1:**
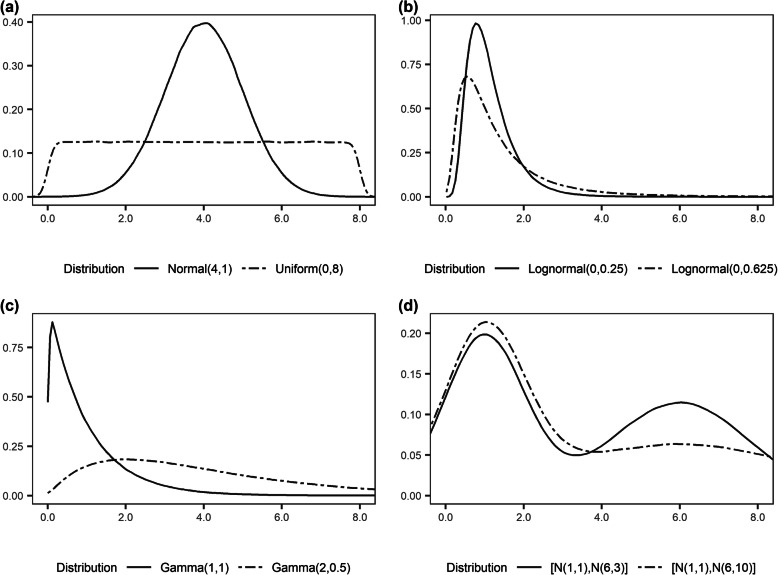
Distributions used for covariate *X*. (**a**) symmetric distributions (normal and uniform), (**b**) lognormal distributions, (**c**) gamma distributions, (**d**) bimodal distributions (mixture of two normal distributions). The panels display the kernel densities based on 1 million observations randomly sampled from each distribution. $\left[\mathrm{N}\left({\mu}_1,{\sigma}_1^2\right),\mathrm{N}\left({\mu}_1,{\sigma}_1^2\right)\right]$ represents a homogeneous mixture of 50% $\mathrm{Normal}\left({\mu}_1,{\sigma}_1^2\right)$ and 50% $\mathrm{Normal}\left({\mu}_1,{\sigma}_1^2\right)$. For figures with boxplots, the top and bottom 0.025 percentiles were truncated to avoid extreme values in order to facilitate the visual comparison of the boxplots

A total of 1000 datasets of 500 observations (where the dependent variable *Y* was continuous) or 1000 observations (where *Y* was binary) were generated for each of the distributions in four scenarios as binary dependent variables usually provide less information for estimating parameters than do continuous dependent variables. The four scenarios comprised of 2 × 2 combinations where *Y* was either continuous or binary, and whether *Y* has a quadratic relationship with the independent variable *X* or its relationship with *X* was modified by a normally distributed variable *Z*. In each scenario, *X* was generated from one of the following eight distributions (Fig. 1):

(1) Normal(4, 1),

(2) Uniform(0, 8),

(3) Lognormal(0, 0.25), Lognormal(0, 0.625),

(4) Gamma(1, 1), Gamma(2, 0.5),

(5) [N(1, 1), N(6, 3)], [N(1, 1), N(6, 10)] (1:1 normal mixtures).

The specific relationships among the variables in each scenario were as follow:

(1) a linear regression with quadratic term:

*Y* = *β*_0_ + *β*_1_*X*_1_ + *β*_2_*X*_2_ + *ε* = 2 + 2*X* + *X*^2^ + *ε*, *ε*~Normal(0, 1.)

(2) a logistic regression with quadratic term:

$$ Y\sim \mathrm{Binomial}\left(1,\pi \right) $$

$$ \mathrm{logit}\left(\pi \right)={\beta}_0+{\beta}_1{X}_1+{\beta}_2{X}_2=-1.2+0.1X+0.05{X}^2 $$

(3) a linear regression with interaction term:

*Y* = *β*_0_ + *β*_1_*X*_1_ + *β*_2_*X*_2_ + *β*_3_*X*_3_ = 2 + *X* + *XZ* + *Z* + *ε*, *ε*~Normal(0, 1),

$$ Z\sim \mathrm{Normal}\left(4,2\right) $$

(4) a logistic regression with interaction term:

$$ Y\sim \mathrm{Binomial}\left(1,\pi \right) $$

$$ \mathrm{logit}\left(\pi \right)={\beta}_0+{\beta}_1{X}_1+{\beta}_2{X}_2+{\beta}_3{X}_3=-2+0.5X-0.0625 XZ+0.25Z $$

$$ Z\sim \mathrm{Normal}\left(4,2\right) $$

In scenario 2, we set *β*_0_ =  − 1.2 so that the probability of *Y* = 1 is *π* = 0.5 when *X* = 4, and *β*_1_ = 2*β*_2_ = 0.1, corresponding to a log odds ratio of 1 for *X* = 5 versus *X* = 3 (plus or minus one standard deviation (SD) when *X*~Normal(4, 1)). The observations with *Y* = 1 can vary in proportions across the different distributions for *X*. In scenario 4, with *β*_0_ =  − 2, the probability of *Y* = 1 is *π* = 0.5 when *X* = 4; *β*_1_ = 0.5, *β*_2_ =  − 0.625 and *β*_3_ = 0.25, corresponding to a log odds ratio of 0.5 for *X* = 5 versus *X* = 3 with *Z* = 4, and a log odds ratio of 0.5 for *X* = 5, *Z* = 6 versus *X* = 3, *Z* = 2.

#### Amputation

Generally, missing data problems can be classified into three categories [13]. When data are MCAR, the probability of being missing is the same for all cases. When data are MAR, the probability of being missing is only related to the observed data. If neither MCAR nor MAR holds, then data are missing not at random (MNAR). While MCAR is simple to consider, most of the missing data methods are designed to address the MAR assumption. In this study, we primarily report situations when data are MAR and refer the reader to supplemental results when data are MCAR. During amputation missing values were introduced into the simulated complete datasets using the amputation function implemented in R by Schouten et al. [14] Specifically, MCAR was introduced by setting the probability of *X* being missing to 25% across observations. MAR was introduced by setting the probability of *X* being missing to 25% according to a standard right-tailed logistic function in *Y*, thus the probability of *X* being missing is higher for observations with higher values of *Y*. Missing values were only generated for *X* (i.e., *Y* and *Z* were kept intact), but its corresponding quadratic or interaction term would also be missing whenever *X* was missing.

#### Imputation

For each amputated dataset, the missing values were imputed by three different imputation methods: PMM, and two RF-based imputation methods, missForest and CALIBERrfimpute as described above.

#### Analysis

Linear or logistic regression was performed on each imputed dataset by regressing *Y* on the other variables in the dataset without knowledge of the presence of quadratic or interaction terms. The three imputation methods were first compared in terms of their accuracy of the imputed values using

(i) the normalized root mean squared error (NRMSE) [15],

$$ \sqrt{\frac{\mathrm{mean}\left({\left({\mathbf{X}}_{\mathrm{true}}-{\mathbf{X}}_{\mathrm{imp}}\right)}^2\right)}{\operatorname{var}\left({\mathbf{X}}_{\mathrm{true}}\right)}}, $$

where ***X***_true_ and ***X***_imp_ are the true and imputed data matrix, respectively, and the mean and variance are computed only over the missing values; and

(ii) relative bias for the mean of the imputed variable:

$$ \frac{\mathrm{mean}\left({V}_{\mathrm{imp}}\right)}{\mathrm{mean}\left({V}_{\mathrm{true}}\right)}-1, $$

where *V* is either one of the imputed variables (*X*, *X*^2^, or *XZ*) *V*_true_ is the original vector of true values, *V*_imp_ is the data vector after imputation, and the mean was computed over all the data values.

Second, the three imputation methods were compared in terms of their accuracy in estimating the coefficients in the linear and logistic regression models using

(i) the relative bias of the coefficient estimate,

$\left({\hat{\beta}}_p-{\beta}_p\right)/{\beta}_p$, *p*= 1 or 2 for imputed variables; and

(ii) the coverage of their 95% confidence intervals.

Third, the three imputation methods were compared in terms of their accuracy in prediction using Lin’s concordance correlation coefficient (CCC) [16] for agreement with the prediction using the true model. The predictions were made from regression models estimated from imputed datasets and the corresponding true model using 1000 new datasets of 500 observations (for continuous outcome) or 1000 observations (for binary outcome) generated as described in the Data Generation section. Lin’s coefficient increases towards one as the predicted values from models estimated using imputed data have better agreement with the predictions from the true model.

An imputation method with superior performance can be generally characterized by smaller NRMSE, relative bias of imputation mean closer to zero, relative bias of regression coefficient estimate closer to zero, coverage closer to nominal coverage probability, and a CCC nearer towards one.

## Results

### Accuracy of imputed variables

#### NRMSE value

Overall, missForest had the smallest NRMSE (mean = 0.39, 0.88, 0.26, 0.79, for scenarios 1 to 4) compared to CALIBERrfimpute (mean = 0.35, 0.98, 0.34, 0.92) and PMM (mean = 0.64, 1.06, 0.48, 1.06) uniformly across all eight distributions except for scenario 1 where CALIBERrfimpute outperformed missForest (Fig. S1).

#### Bias of variable estimates

**Fig. 2:**
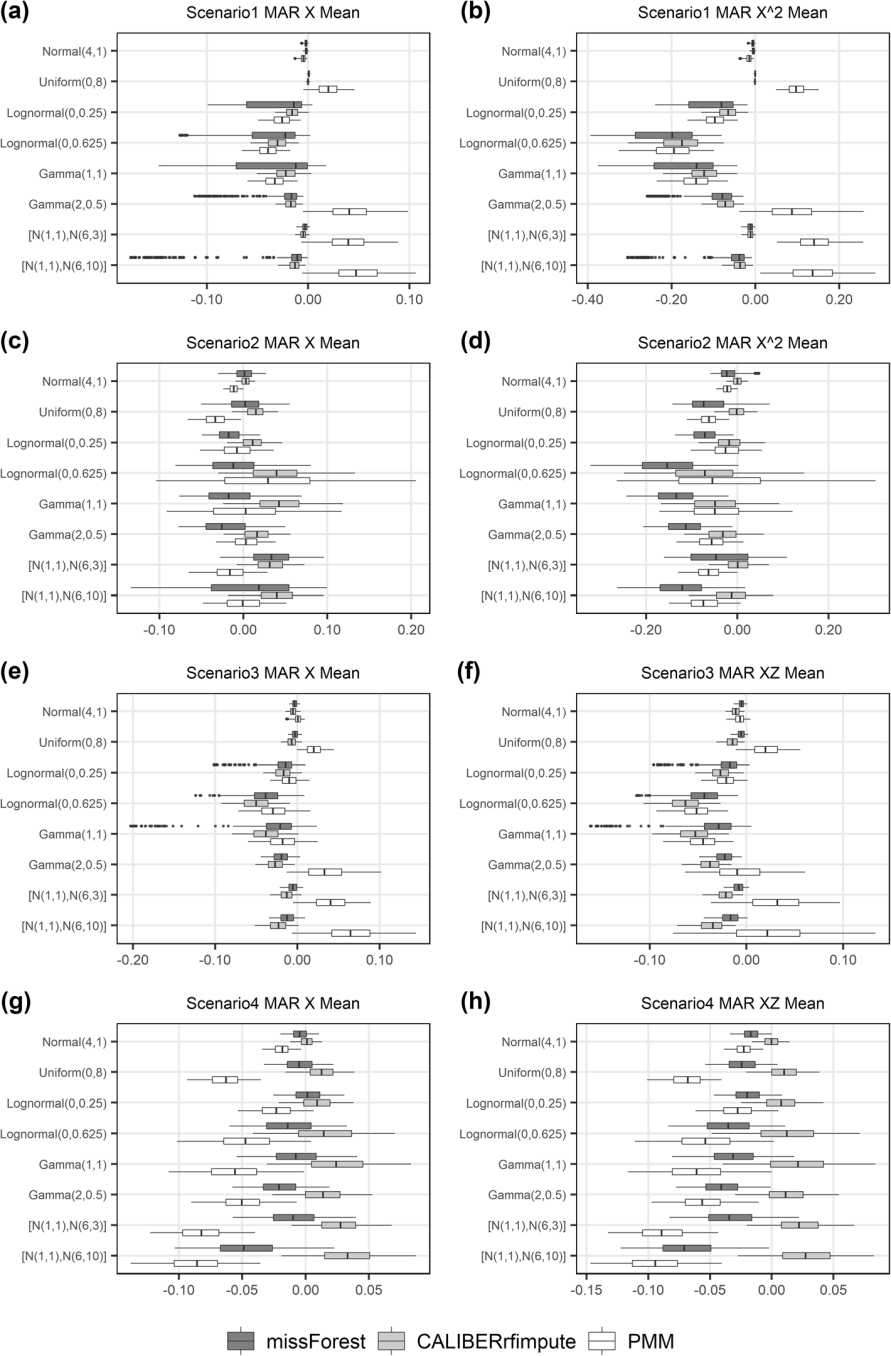
Relative bias of the estimated mean of imputed variables for MAR data

When estimating the mean of *X* across the eight distributions (Fig. 2), missForest on average gave relative biases of 2.0, 1.3, 1.7, 1.4%, compared to 1.4, 2.5, 2.3, 1.7% in CALIBERrfimpute, 3.2, 1.4, 2.7, 5.3% in PMM for scenarios 1 through 4, respectively. (To be concise, we report in the text the mean of the absolute values of the mean relative bias for each distribution when summarizing the relative bias across the eight distributions.) MissForest had the smallest bias except for scenario 1. When estimating the mean of *X*^2^ or *XZ* across the eight distributions, missForest gave relative biases of 8.3, 8.9, 2.1, 3.4% compared to 6.3, 2.3, 3.4, 1.4% in CALIBERrfimpute, 11.6, 4.8, 2.6, 5.9% in PMM for scenarios 1 through 4, respectively. CALIBERrfimpute outperformed missForest except for scenario 3, where missForest had smaller bias. MissForest can produce more biased results for non-normal data.

### Accuracy of regression estimates

#### Bias of regression coefficient estimates

**Fig. 3:**
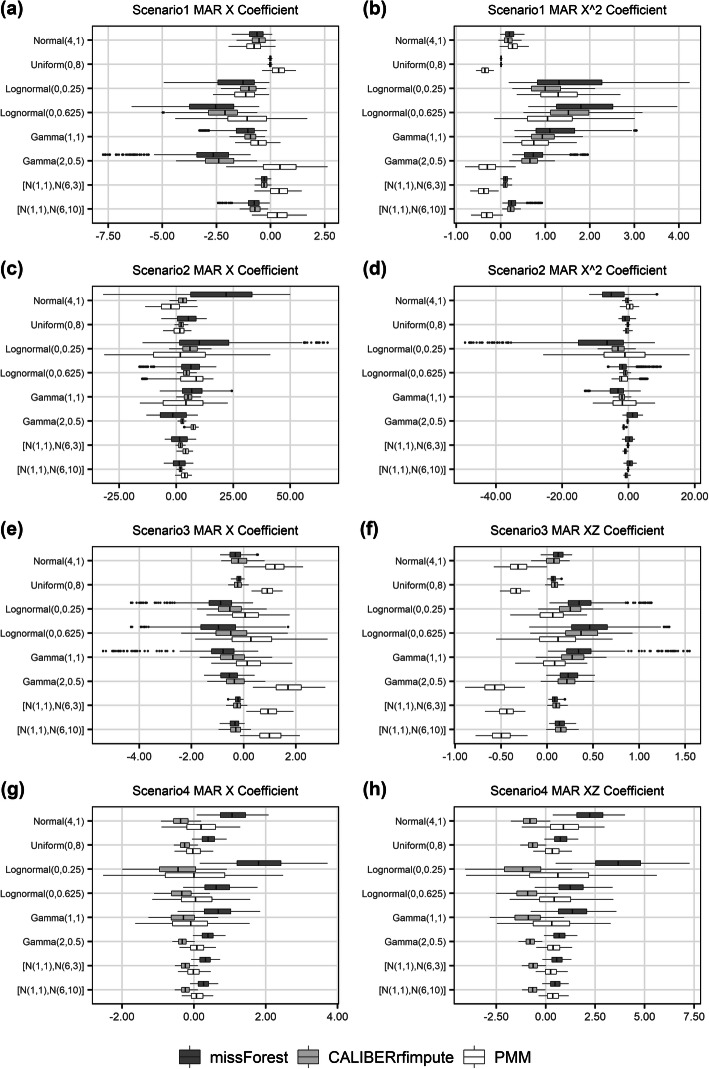
Relative bias of the estimated regression coefficient of imputed variables for MAR data

In contrast to estimating the characteristics of the imputed variables, the ability of the imputation methods to estimate regression coefficients was much poorer (Fig. 3), especially for logistic regressions. When estimating the regression coefficient of *X* across the eight distributions, missForest on average gave relative biases of 133.1, 653.3, 58.6, 70.4%, compared to 104.3, 335.8, 32.9, 31.3% in CALIBERrfimpute, 64.2, 371.0, 80.4, 7.7% in PMM for scenarios 1 through 4, respectively. PMM out-performed RF-based methods in both scenarios 1 and 4. When estimating the mean of *X*^2^ or *XZ*, missForest gave relative biases of 78.7, 252.7, 23.9, 138.1%, compared to 59.9, 94.6, 18.8, 82.3% in CALIBERrfimpute, 61.1, 101.0, 30.0, 47.0% in PMM for scenarios 1 through 4, respectively. PMM outperformed RF-based methods in scenario 4. For RF-based imputation, the bias of the estimated means and the bias of their corresponding estimated regression coefficients can be strongly correlated. Likewise, as in the case for estimating means, missForest can produce more biased results for non-normal data.

#### Coverage of 95% confidence intervals

**Fig. 4:**
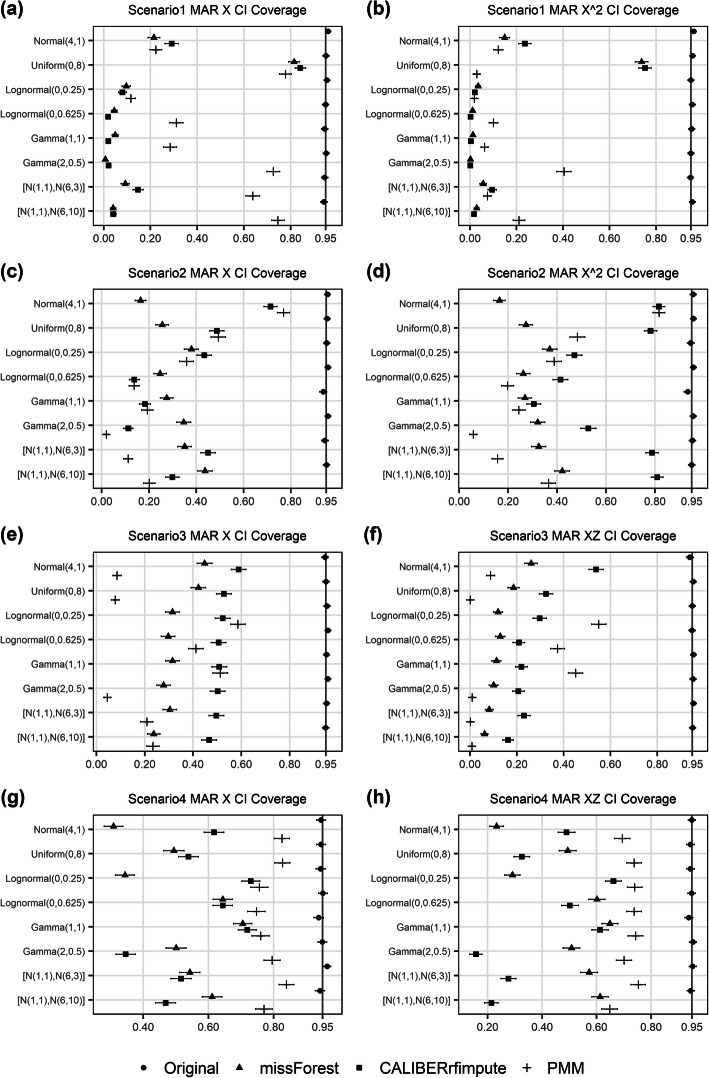
Coverage of 95% confidence intervals (with binomial proportion confidence intervals) of the estimated regression coefficients of imputed variables for MAR data

The coverages of the confidence intervals were uniformly poor for all three imputation methods. When estimating the regression coefficient of *X* across the eight distributions (Fig. 4), missForest gave coverages of 17.0, 30.7, 32.7, 51.9%, compared to 18.3, 35.2, 51.5, 57.3% in CALIBERrfimpute, 47.8, 28.5, 27.0, 79.0% in PMM, and 95.1, 95.1, 95.3, 94.7% in the original non-missing data, for scenarios 1 through 4, respectively. When estimating the regression coefficient of *X*^2^ or *XZ*, missForest gave coverages of 12.9, 30.1, 13.2, 49.5%, compared to 14.2, 61.4, 27.4, 40.5% in CALIBERrfimpute, 12.8, 33.9, 18.5, 71.9% in PMM, and 95.4, 95.1, 95.1, 94.8% in the original non-missing data, for scenarios 1 through 4, respectively.

### Accuracy of regression model predictions

**Fig. 5:**
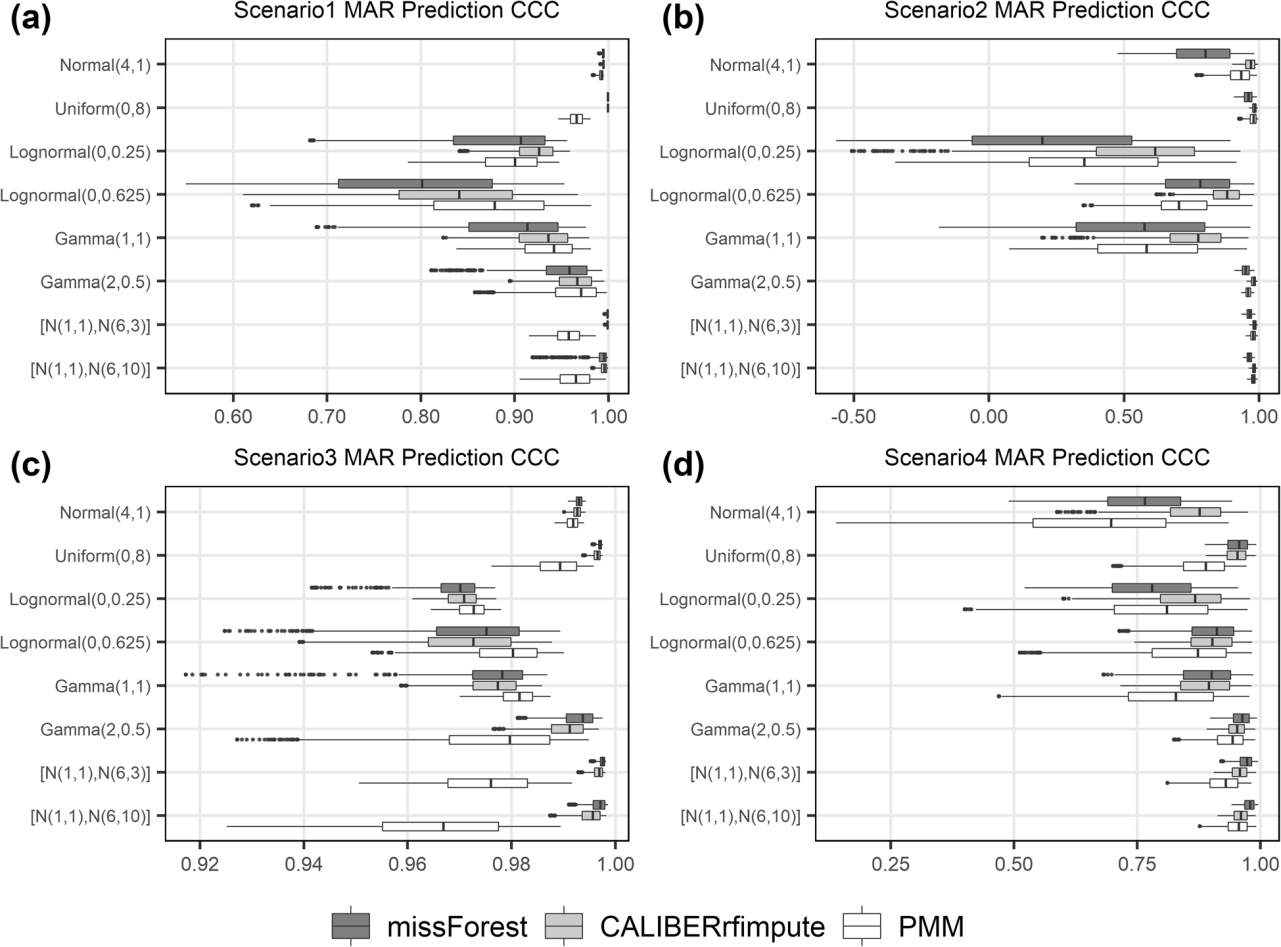
Lin’s concordance correlation coefficient (CCC) from predictions using models estimated from imputed MAR data

When predicting new data (Fig. 5), missForest on average gave CCCs of 0.93, 0.76, 0.99, 0.89, compared to 0.95, 0.88, 0.99, 0.91 in CALIBERrfimpute, and 0.94, 0.81, 0.98, 0.84 in PMM, for scenarios 1 through 4, respectively. CALIBERrfimpute imputation had the highest prediction accuracy, but for logistic regression the agreement is poorer due to biased coefficient estimates.

### Case study

To explore the imputation performance of the aforementioned methods in real datasets, a longitudinal study on patients with HCC was examined [17]. It should be noted that the analysis presented here was to investigate the imputation methods, and is not intended as a definitive analysis of the data. Two baseline covariates, serum alpha-fetoprotein (AFP) level (IU/ml) and diameter of main tumor (mm), were considered important prognostic factors for HCC [18, 19], and thus were standardized and used to predict survival at 12 months after baseline using logistic regression. The complete dataset consisting of 1897 patients were amputed like what was done in the Amputation section. except here both covariates were MCAR, or MAR according to the outcome. About 25% of the observations were missing, and at most one covariate value was missing for a single observation. Table 1 shows that more biased estimates of regression coefficients can be observed in missForest for MAR data and even for MCAR data. These results also confirm the previous simulation results that RF-based imputation does not outperform PMM in the presence of a logistic regression relationship.

**Table 1:** Parameter estimates of the regression from the case study

Method	Intercept		Regression coefficient for AFP		Regression coefficient for tumor diameter	
	Estimate (95% CI)	*p*-value	Estimate (95% CI)	*p*-value	Estimate (95% CI)	*p*-value
Original data	0.54 (0.43, 0.64)	< 0.001	−0.20 (− 0.31, − 0.10)	< 0.001	− 0.65 (− 0.76,-0.54)	< 0.001
MAR data						
PMM	0.55 (0.45, 0.65)	< 0.001	− 0.19 (− 0.29, − 0.09)	< 0.001	−0.66 (− 0.77, − 0.56)	< 0.001
missForest	0.54 (0.44, 0.64)	< 0.001	−0.17 (− 0.28, − 0.07)	0.002	−0.74 (− 0.85, − 0.62)	< 0.001
CALIBERrfimpute	0.54 (0.44, 0.64)	< 0.001	−0.19 (− 0.29, − 0.09)	< 0.001	−0.66 (− 0.77, − 0.56)	< 0.001
MCAR data						
PMM	0.54 (0.44, 0.64)	< 0.001	−0.20 (− 0.31, − 0.10)	< 0.001	−0.66 (− 0.77, − 0.56)	< 0.001
missForest	0.54 (0.44, 0.64)	< 0.001	−0.21 (− 0.32, − 0.10)	< 0.001	−0.72 (− 0.84, − 0.61)	< 0.001
CALIBERrfimpute	0.55 (0.45, 0.65)	< 0.001	−0.22 (− 0.33, − 0.12)	< 0.001	−0.68 (− 0.79, − 0.58)	< 0.001

*Results were obtained from logistic regression

*CI* confidence interval, *MCAR* missing completely at random, *MAR* missing at random

## Discussion

MissForest has been reported to “successfully handle missing values, particularly in datasets including different types of variables” [2] (p.112). Waljee et al. further concluded that it “is a highly accurate method of imputation for missing laboratory data and outperforms other common imputation techniques” [6] (p.1). These two studies focused on MCAR data, and while other researchers have examined missForest in the presence of MAR [4] and even MNAR data [5], all of these research judged missForest in terms of its predictive accuracy. However, an imputation method that simply imputes missing values by minimizing prediction error can be problematic since it does not try to recover the joint distribution of the data and thus can result in biased parameter estimates. Therefore, other measures of imputation accuracy; i.e., bias of imputation mean and regression coefficient estimate, and coverage would be more important and relevant when considering all the variables together.

The simulations showed that missForest typically had the lowest NRMSE and smaller bias when estimating the mean of the imputed variables compared to PMM. However, missForest often under-estimated the standard deviation of the imputed variables, suggesting that subsequent significance testing would result in elevated false positive error rates, and this is because missForest simply used the conditional mean for imputation. Yet, the more critical issue is that good predictive accuracy of imputed values does not necessarily imply better accuracy in estimating the relationships involving the imputed variables. For MAR data, all the imputation approaches were considerably biased when estimating regression coefficients. MissForest was shown to produce many-fold more biased regression coefficient estimates than PMM, and its inferior performance often appeared when the imputed variable was highly skewed in nonlinear models or when there was an interaction term. (This is consistent with the biased Cox regression coefficient estimates in missForest reported by Shah et al. [3].) Under such circumstances, CALIBERrfimpute can often outperform missForest, but its bias was still substantial. Due to single imputation and biased regression coefficient estimates, coverages of confidence intervals were far from nominal coverage. This was also the case even for MCAR data (refer to Supplementary Information for results). For MCAR data, RF-based imputation showed higher accuracy than PMM for mean and regression coefficient estimates except for estimating regression coefficients in scenario 4.

**Fig. 6:**
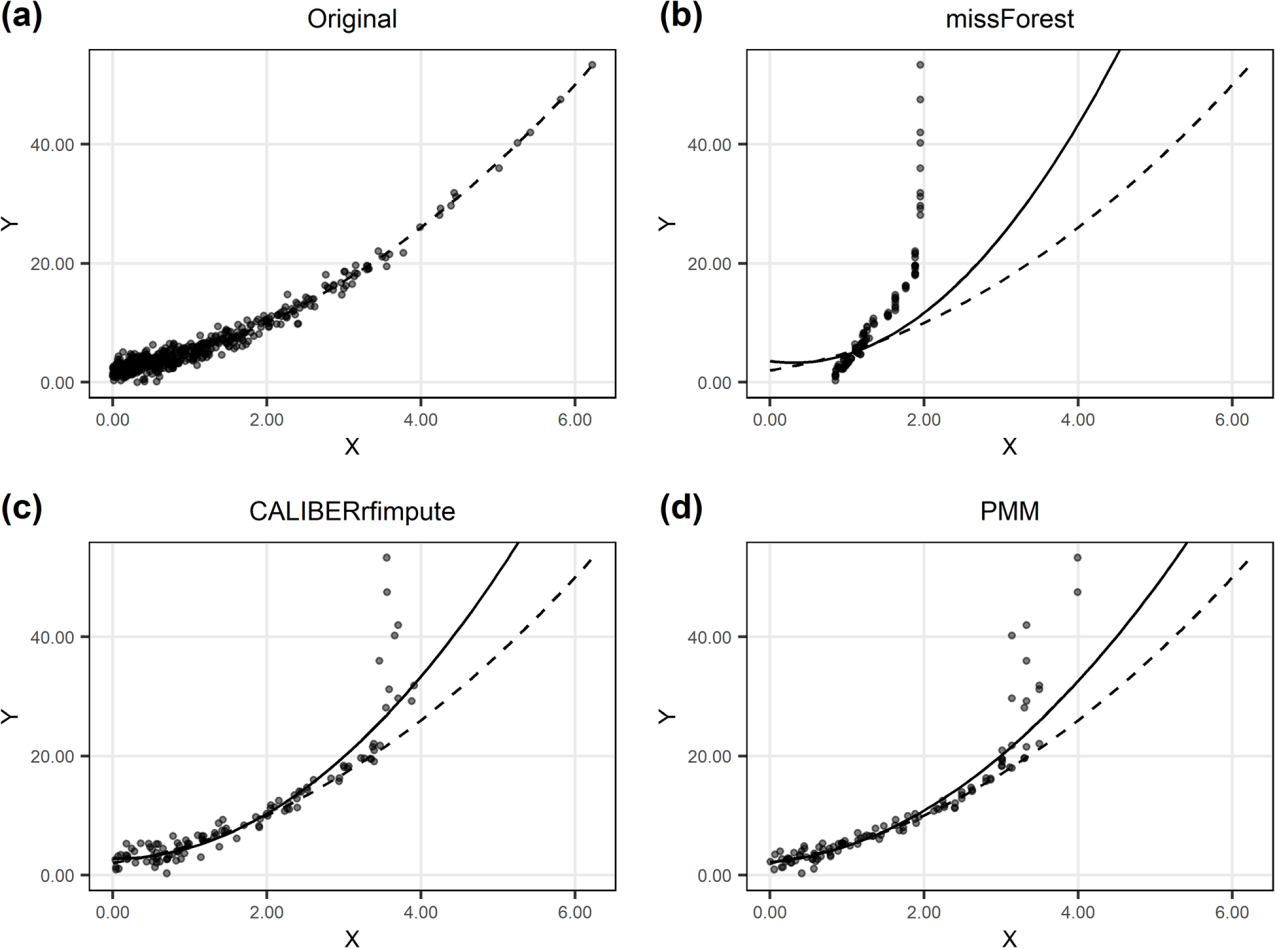
Scatter plot of *Y* versus *X* from imputation results of a randomly selected dataset when *X*~Gamma(1, 1) in scenario 1 for MAR data. Dashed line is for the true model, solid line is for the estimated model using imputed data. Only imputed observations were shown for direct comparison between imputation methods

The conditional mean prediction from missForest is a weighted average of the imputed variable’s observed values, and when data are outcome-dependent MAR this can result in information loss near both ends of the variable range and severely biased regression estimates, especially when variables are highly skewed and non-linearity is present (Fig. 6). While CALIBERrfimpute can overcome this issue to a certain extent by taking the prediction errors of RF into consideration and drawing imputations from the conditional distributions, it cannot fundamentally recover the information loss due to the sparseness and high leverage data points. Such truncation in the variable range is also apparent for PMM, although to a lesser degree.

The simulations only considered (generalized) linear models so favorable imputation results can be achieved by PMM in certain scenarios as PMM is essentially linear-model-based, but for data with complex relationships PMM may not have such advantages over RF-based methods. Since missForest was originally designed to be a single imputation method, this study used single imputation for CALIBERrfimpute and PMM to facilitate comparison. Therefore, all three imputation methods did not account for the uncertainty from missing data in the analysis. This can be problematic especially when the proportion of missingness is high and may lead to inflated false positive error rates. In practice, multiple imputation should be recommended for considering the uncertainty induced by missing data. The results showed that CALIBERrfimpute can often outperform missForest, indicating that sampling from a conditional distribution can help in the estimation of regression coefficients and variable means even with only single imputation. However, it should be noted that its out-of-bag mean squared error can be influenced by extreme values in the variable under imputation, leading to biased results even when data are MCAR. Therefore, the detection and handling of extreme values when using CALIBERrfimpute should be considered for less biased results.

Eight common distributions and four simple regression models were used to generate 32 simulation settings for examining the accuracy of the imputation methods. The chosen distributions provided a variety of non-normal symmetric, heavy tailed, and skewed distributions, and the regression models considered both non-linearity and interaction coupled with either a continuous or binary outcome. These settings are intended to depict the mixed data types that missForest was believed to handle well. The performance of the imputation methods was weaker for logistic regression models because the binary outcomes provided less information for estimating regression parameters than the continuous outcomes, and the estimation of the log odds ratios was also more sensitive to inaccuracies in the imputed variables.

The simulations generated a realistic proportion of missing data, and increasing the proportion of missing data to 50% did not materially alter the main findings. A straightforward MAR data pattern was introduced where the probability of *X* being missing was dependent on larger observed outcome values. Although MNAR data cannot be ruled out in practice, we focused on the MAR mechanism to highlight how RF-based imputation algorithms can be problematic even though multiple imputation was designed to address MAR data. Another limitation is that *Y* was assumed to be free of missing values since we wanted to use a relatively simple MAR mechanism to examine how different distributions of *X* can impact the imputation of its missing values. MissForest also performed poorly when estimating regression relationships where the MAR mechanism was additionally dependent on other covariates [3], suggesting that outcome-dependent MAR is a sufficient reason for its deficiency.

The number of trees built for RF models was set to 10 to limit bias and compared to PMM operating under their default parameters. The iteration number for MICE-based imputation was set to five as recommended and for a faster computational speed. But in practice the influence of tuning parameters on imputation accuracy and computational burden still requires additional study, and parameter searching may be necessary for complex datasets to attain better imputation accuracy. For example, it is unclear how different data structures and missingness may impact missForest’s stopping criterion, and users should monitor whether missForest stopped because it reached the maximum number of iterations as this may indicate a slow or problematic convergence.

For all three imputation methods, an inclusive imputation model that included the outcome was used. In situations where the imputation model fails to account for variables that impact the occurrence of missing data or does not accurately capture the relationship of the variables, non-parametric imputation methods may fare better than parametric imputation methods and further study is warranted.

## Conclusions

The results of the simulation experiments and case study present evidence that although RF-based imputation can have good predictive accuracy, it may also lead to severely biased inference when the imputed variables are used in subsequent regression analyses. Missforest has the characteristic that its predicted value does not extend beyond the range of the imputed variable’s observed values but this can lead to underperformance with outcome-dependent MAR and highly skewed data. CALIBERrfimpute can slightly alleviate this problem by sampling from the RF predicted value’s conditional distribution but regression estimates still have substantial bias and poor confidence interval coverage. Therefore, RF-based imputation and especially missForest should not be indiscriminately used as a panacea for imputing missing data. Once again it proves that a correct analysis requires a careful critique of the missing data mechanism and the inter-relationships between the variables in the data.

## Supplementary information

**Additional file 1: Appendix S1.** Distributions and functions used for simulation studies. **Appendix S2.** Additional results for MAR data. **Appendix S3.** Results for MCAR data.

**Additional file 2: Figure S1.** NRMSE value for MAR data.

**Additional file 3: Figure S2.** Standard deviation of imputed variables for MAR data.

**Additional file 4: Figure S3.** Width of 95% confidence intervals of the estimated regression coefficients of imputed variables for MAR data.

**Additional file 5: Figure S4.** NRMSE value for MCAR data.

**Additional file 6: Figure S5.** Relative bias of the estimated mean of imputed variables for MCAR data.

**Additional file 7: Figure S6.** Standard deviation of imputed variables for MCAR data.

**Additional file 8: Figure S7.** Relative bias of the estimated regression coefficient of imputed variables for MCAR data.

**Additional file 9: Figure S8.** Width of 95% confidence intervals of the estimated regression coefficients of imputed variables for MCAR data.

**Additional file 10: Figure S9.** Coverage of 95% confidence intervals (with binomial proportion confidence intervals) of the estimated regression coefficients of imputed variables for MCAR data.

**Additional file 11: Figure S10.** Lin’s concordance correlation coefficient (CCC) from predictions using models estimated from imputed MCAR data.

## Data Availability

Further details of the simulation studies are provided in the supplementary information, and software packages used in this study are publicly available on the Comprehensive R Archive Network (CRAN), https://cran.r-project.org. The dataset supporting the conclusions of this article is available in the Dryad Digital Repository, https://datadryad.org (doi:10.5061/dryad.pd44k8r).

## Abbreviations

CCC: Concordance correlation coefficient
HCC: Hepatocellular carcinoma
kNN: K-nearest neighbors
MAR: Missing at random
MCAR: Missing completely at random
MICE: Multivariate imputation using chained equations
MNAR: Missing not at random
NRMSE: Normalized root mean squared error
PMM: Predictive mean matching
RF: Random forests
SD: Standard deviation
