# Supplementary material for: Accuracy of random-forest-based imputation of missing data in the presence of non-normality, non-linearity, and interaction
Source: BMC Med Res Methodol. 2020 Jul 25;20:199. doi: 10.1186/s12874-020-01080-1 (PMC7382855; doi:10.1186/s12874-020-01080-1)
Supplement: Supplementary file 1 — Additional file 1: Appendix S1. Distributions and functions used for simulation studies. Appendix S2. Additional results for MAR data. Appendix S3. Results for MCAR data. [file 12874_2020_1080_MOESM1_ESM.docx]

Supplementary Information

Shangzhi Hong, Henry S. Lynn^*^

# S1 Distributions and functions used for simulation studies

## S1.1 Mixture of normal distributions

For a finite set of normal probability density functions $f_{1}\left( x \right),\cdots,f_{n}\left( x \right)$, and corresponding weights of the probability densities $w_{1},\cdots,w_{n}$ such that $w_{i}\geq0$, and $\sum w_{i}=1$, then a mixture of normal distributions is represented by the probability density:

$$f\left( X \right)=\sum_{i=1}^{n} w_{i}f_{i}\left( x \right)$$

where $f_{i}\left( x \right)\sim\text{Normal}\left( \mu_{i},\sigma_{i}^{2} \right)$, and in the simulation of bimodal normal distributions in this study the parameters are: $n=2$, and $w_{1}=w_{2}=0.5$.

## S1.2 Lognormal distributions

A random variable $X$ is lognormally distributed with parameters $\mu$ and $\sigma^{2}$, i.e. $X\sim\text{Lognormal}\left( \mu,\sigma^{2} \right)$ , when a random variable $Z=\log\left( X \right)$ is normally distributed with mean $\mu$ and variance $\sigma^{2}$, i.e. $Z\sim\text{Normal}\left( \mu,\sigma^{2} \right)$.

# S2 Additional results for missing at random data

## S2.1 Standard deviation

All imputation methods can lead to decreased standard deviation of the imputed variables (Fig. S2), except for PMM in a few cases. CALIBERrfimpute can lead to closer SD values to the original non-missing data than missForest in certain cases.

## S2.2 Width of 95% confidence intervals

When estimating the regression coefficient of $X$ across the eight distributions (Fig. S3), missForest on average gave confidence interval widths of 1.98, 0.99, 0.81, 0.71, compared to 1.76, 0.55, 0.95, 0.45 in CALIBERrfimpute, 2.89, 0.83, 1.07, 0.66 in PMM, 0.44, 0.78, 0.40, 0.62 in the original non-missing data, for scenarios 1 through 4, respectively. When estimating the regression coefficient of $X^{2}$ or $XZ$ across the eight distributions, missForest on average gave confidence interval widths of 0.41, 0.20, 0.20, 0.16, compared to 0.34, 0.11, 0.24, 0.10 in CALIBERrfimpute, 0.47, 0.17, 0.27, 0.15 in PMM, 0.08, 0.15, 0.09, 0.14 in the original non-missing data, for scenarios 1 through 4, respectively. While missForest can lead to increased width of confidence intervals, CALIBERrfimpute can lead to decreased width of confidence intervals for the imputed variables in scenarios 2 and 4.

# S3 Results for missing completely at random data

## S3.1 Accuracy of imputed variables

### S3.1.1 NRMSE value

Overall, missForest had the smallest NRMSE (mean=0.17, 0.87, 0.15, 0.80, for scenarios 1 to 4) compared to CALIBERrfimpute (mean=0.27, 1.05, 0.30, 0.94) and PMM (mean=0.92, 1.15, 0.56, 1.09) uniformly across all eight distributions (Fig. S4).

### S3.1.2 Bias of variable estimates

When estimating the mean of $X$ across the eight distributions (Fig. S5), missForest on average gave relative biases of 1.6%, 2.1%, 0.8%, 2.5%, compared to 0.7%, 2.7%, 0.5%, 1.8% in CALIBERrfimpute, 3.1%, 0.8%, 2.1%, 3.0% in PMM for scenarios 1 through 4, respectively. When estimating the mean of $X^{2}$ or $XZ$ across the eight distributions, missForest on average gave relative biases of 0.6%, 4.3%, 0.7%, 0.6%, compared to 0.3%, 0.6%, 0.4%, 1.4% in CALIBERrfimpute, 8.9%, 0.7%, 3.5%, 3.4% in PMM for scenarios 1 through 4, respectively. CALIBERrfimpute performed the best except for scenario 2, where PMM had the smallest bias.

### S3.1.3 Standard Deviation

Similar to MAR data, all imputation methods can lead to decreased standard deviation of the imputed variables (Fig. S6), except for PMM in a few cases. CALIBERrfimpute can lead to closer SD values to the original non-missing data than missForest in certain cases.

## S3.2 Accuracy of regression estimates

### S3.2.1 Accuracy of regression estimates

The ability of the imputation methods to estimate regression coefficients was better than that for MAR data, but the overall bias was still considerable for logistic regressions (Fig. S7). When estimating the regression coefficient of $X$ across the eight distributions, missForest on average gave relative biases of 8.0%, 181.8%, 8.1%, 11.8%, compared to 6.0%, 114.0%, 7.6%, 40.6% in CALIBERrfimpute, 50.0%, 257.3%, 25.3%, 2.1% in PMM for scenarios 1 through 4, respectively. When estimating the regression coefficient of $X^{2}$ or $XZ$ across the eight distributions, missForest on average gave relative biases of 5.5%, 79.6%, 4.1%, 19.0%, compared to 2.1%, 16.4%, 1.8%, 88.4% in CALIBERrfimpute, 27.9%, 78.5%, 19.8%, 13.4% in PMM for scenarios 1 through 4, respectively. PMM outperformed RF-based methods in scenario 4.

### S3.2.2 Width of 95% confidence intervals

When estimating the regression coefficient of $X$ across the eight distributions (Fig. S8), missForest on average gave confidence interval widths of 0.59, 0.90, 0.45, 0.66, compared to 0.68, 0.51, 0.59, 0.44 in CALIBERrfimpute, 1.93, 0.74, 0.93, 0.64 in PMM, 0.44, 0.78, 0.40, 0.62 in the original non-missing data, for scenarios 1 through 4, respectively. When estimating the regression coefficient of $X^{2}$ or $XZ$ across the eight distributions, missForest on average gave confidence interval widths of 0.10, 0.17, 0.11, 0.15, compared to 0.11, 0.10, 0.14, 0.10 in CALIBERrfimpute, 0.23, 0.14, 0.22, 0.15 in PMM, 0.08, 0.14, 0.09, 0.14 in the original non-missing data, for scenarios 1 through 4, respectively. While missForest can lead to increased width of confidence intervals, CALIBERrfimpute can lead to decreased width of confidence intervals for the imputed variables in scenarios 2 and 4, similar to that observed for MAR data.

### S3.2.3 Coverage of 95% confidence intervals

The coverage of the estimated regression coefficient confidence intervals was better than that of MAR data, but due to single imputation and biased regression coefficient estimates the coverage was still overall poor. When estimating the regression coefficient of $X$ across the eight distributions (Fig. S9), missForest on average gave coverages of 66.3%, 48.9%, 80.4%, 87.6%, compared to 64.9%, 69.2%, 69.7%, 43.3% in CALIBERrfimpute, 54.1%, 40.1%, 60.5%, 85.1% in PMM, 95.1%, 94.4%, 95.0%, 94.7% in the original non-missing data, for scenarios 1 through 4, respectively. When estimating the regression coefficient of $X^{2}$ or $XZ$ across the eight distributions, missForest on average gave coverages of 61.2%, 45.8%, 63.0%, 89.3%, compared to 58.8%, 79.1%, 66.2%, 33.9% in CALIBERrfimpute, 37.6%, 39.3%, 29.7%, 84.5% in PMM, 95.1%, 94.5%, 94.8%, 94.7% in the original non-missing data, for scenarios 1 through 4, respectively.

## 2.2.4 Accuracy of regression model predictions

The prediction accuracy was similar across different imputation methods. MissForest on average gave CCCs of 0.99, 0.90, 0.99, 0.93, compared to 0.99, 0.95, 0.99, 0.92 in CALIBERrfimpute, 0.94, 0.89, 0.97, 0.92 in PMM, for scenarios 1 through 4, respectively (Fig. S10). Overall, CALIBERrfimpute imputation had the highest prediction accuracy except for scenario 4, where missForest can have a small advantage. For logistic regression, the agreement is poorer due to biased coefficient estimates.
