# Supplementary figures and images for: Accuracy of random-forest-based imputation of missing data in the presence of non-normality, non-linearity, and interaction
Source: BMC Med Res Methodol. 2020 Jul 25;20:199. doi: 10.1186/s12874-020-01080-1 (PMC7382855; doi:10.1186/s12874-020-01080-1)

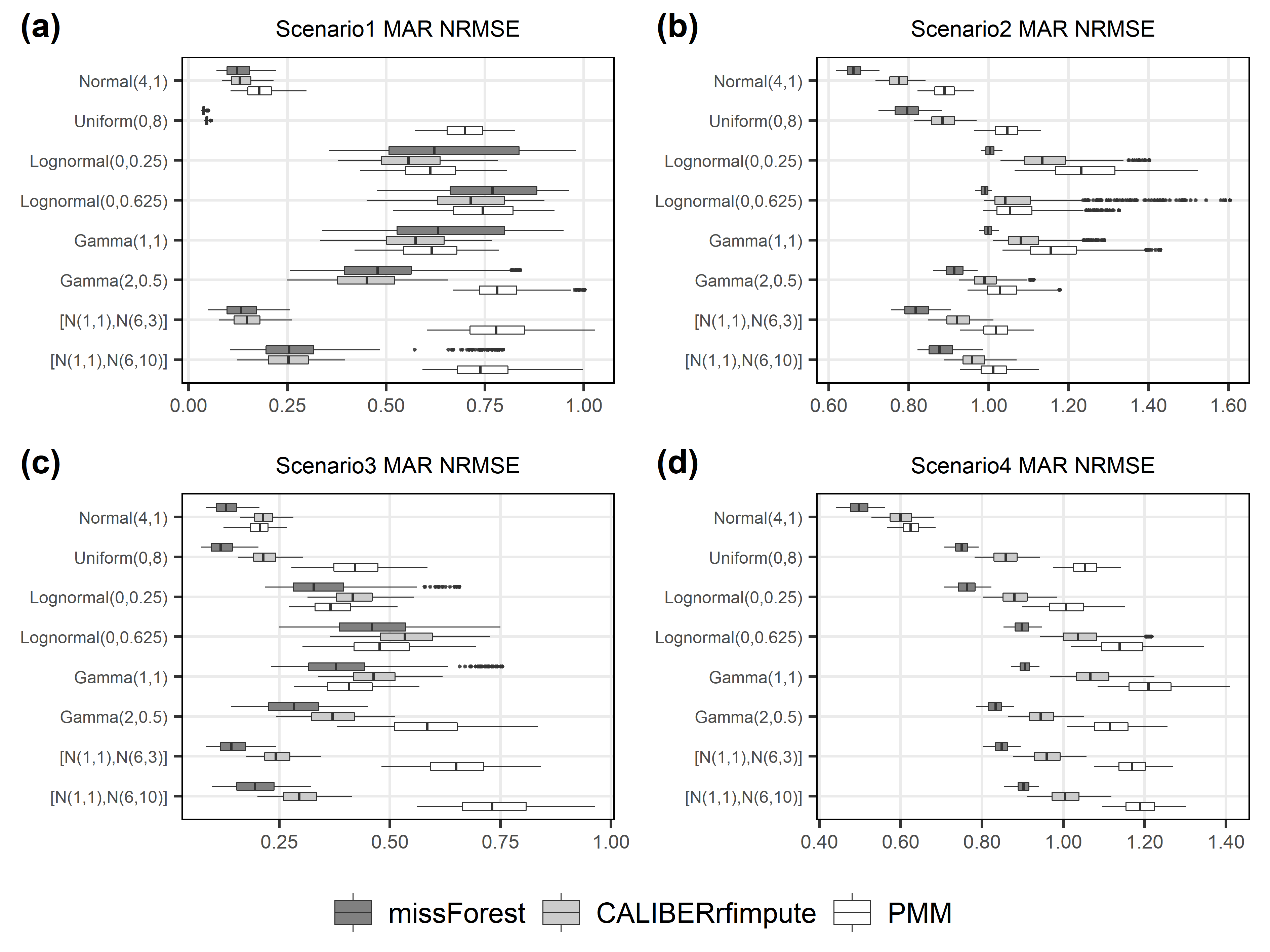

Supplement: Supplementary file 2 — Additional file 2: Figure S1. NRMSE value for MAR data. [file 12874_2020_1080_MOESM2_ESM.tiff]

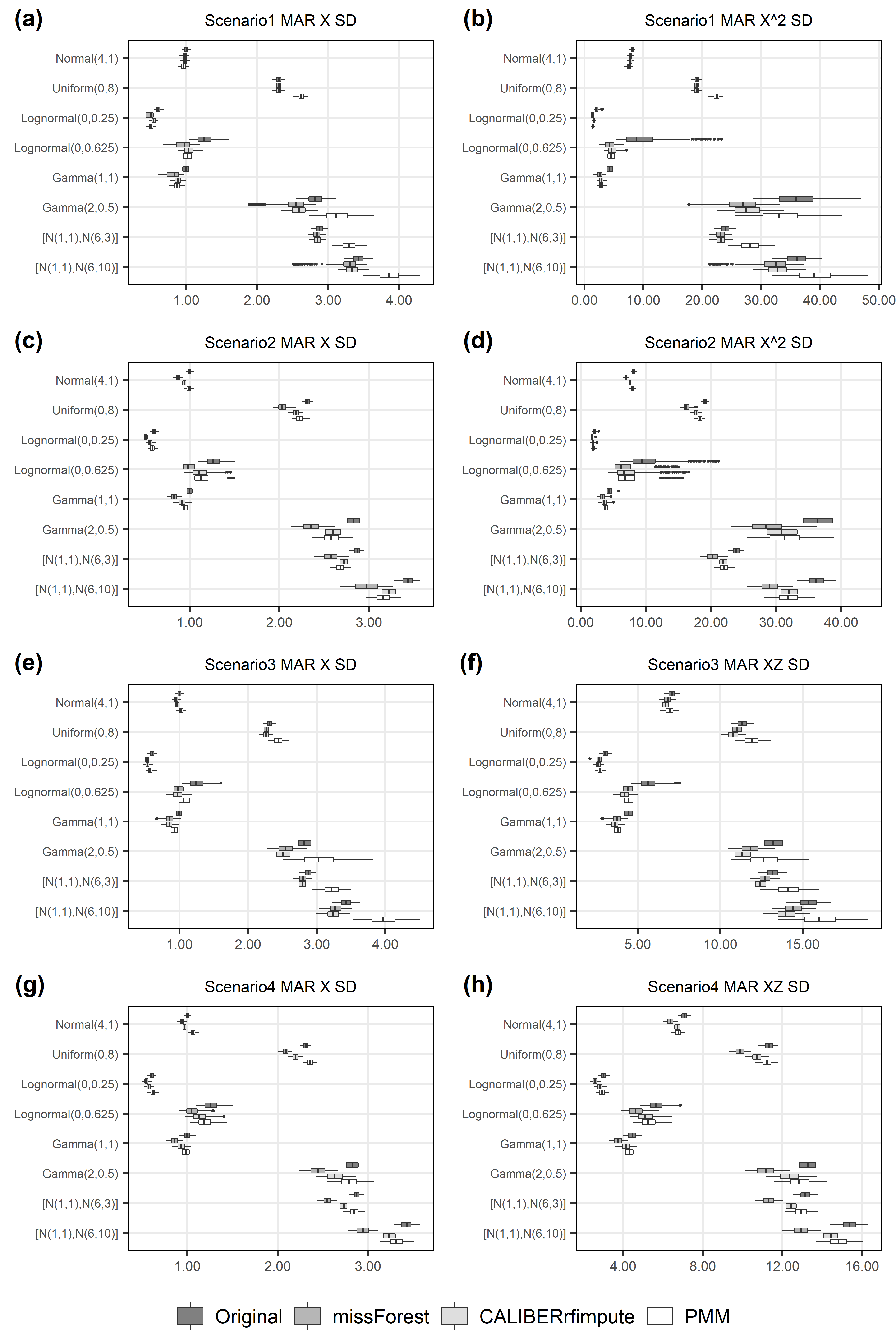

Supplement: Supplementary file 3 — Additional file 3: Figure S2. Standard deviation of imputed variables for MAR data. [file 12874_2020_1080_MOESM3_ESM.tiff]

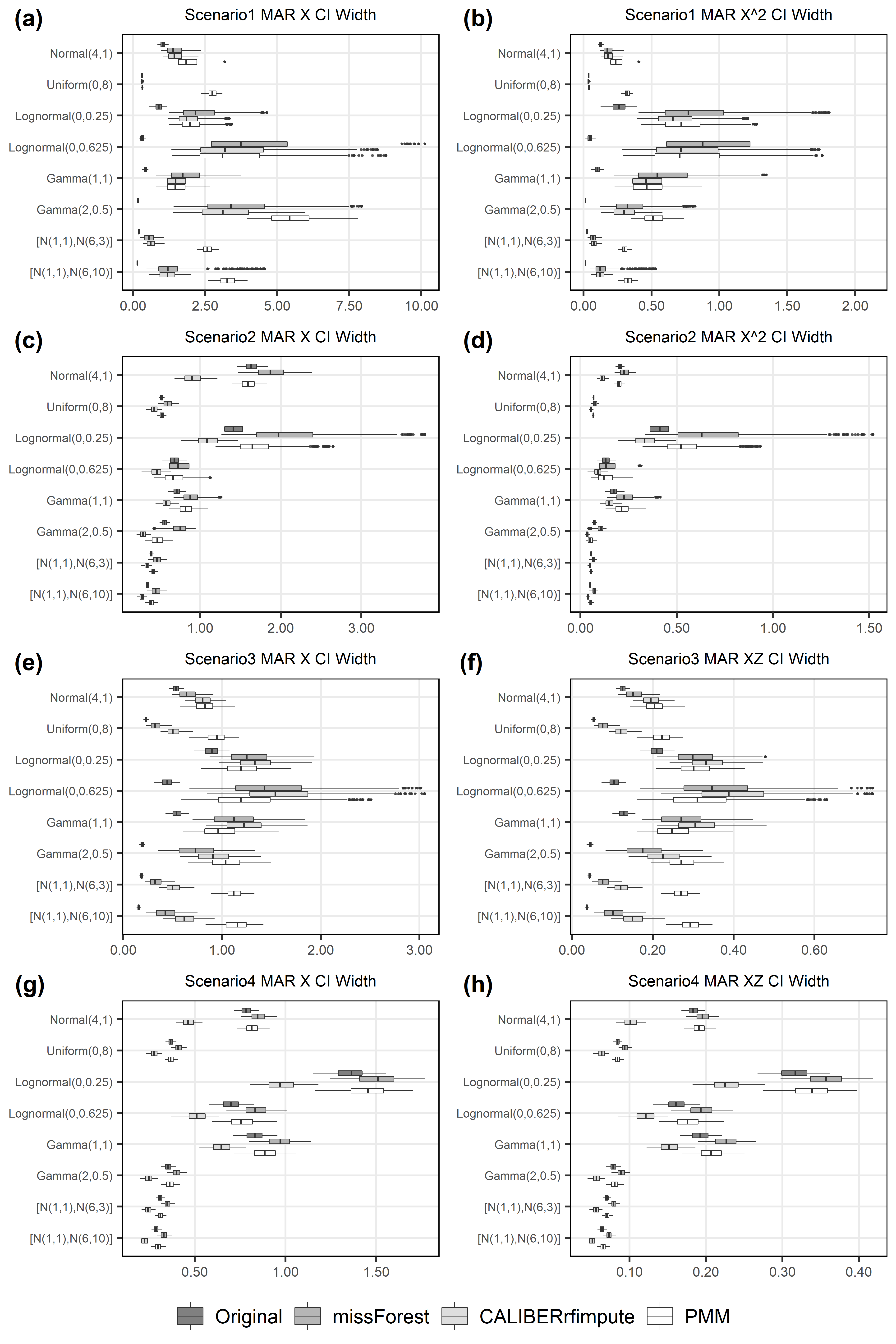

Supplement: Supplementary file 4 — Additional file 4: Figure S3. Width of 95% confidence intervals of the estimated regression coefficients of imputed variables for MAR data. [file 12874_2020_1080_MOESM4_ESM.tiff]

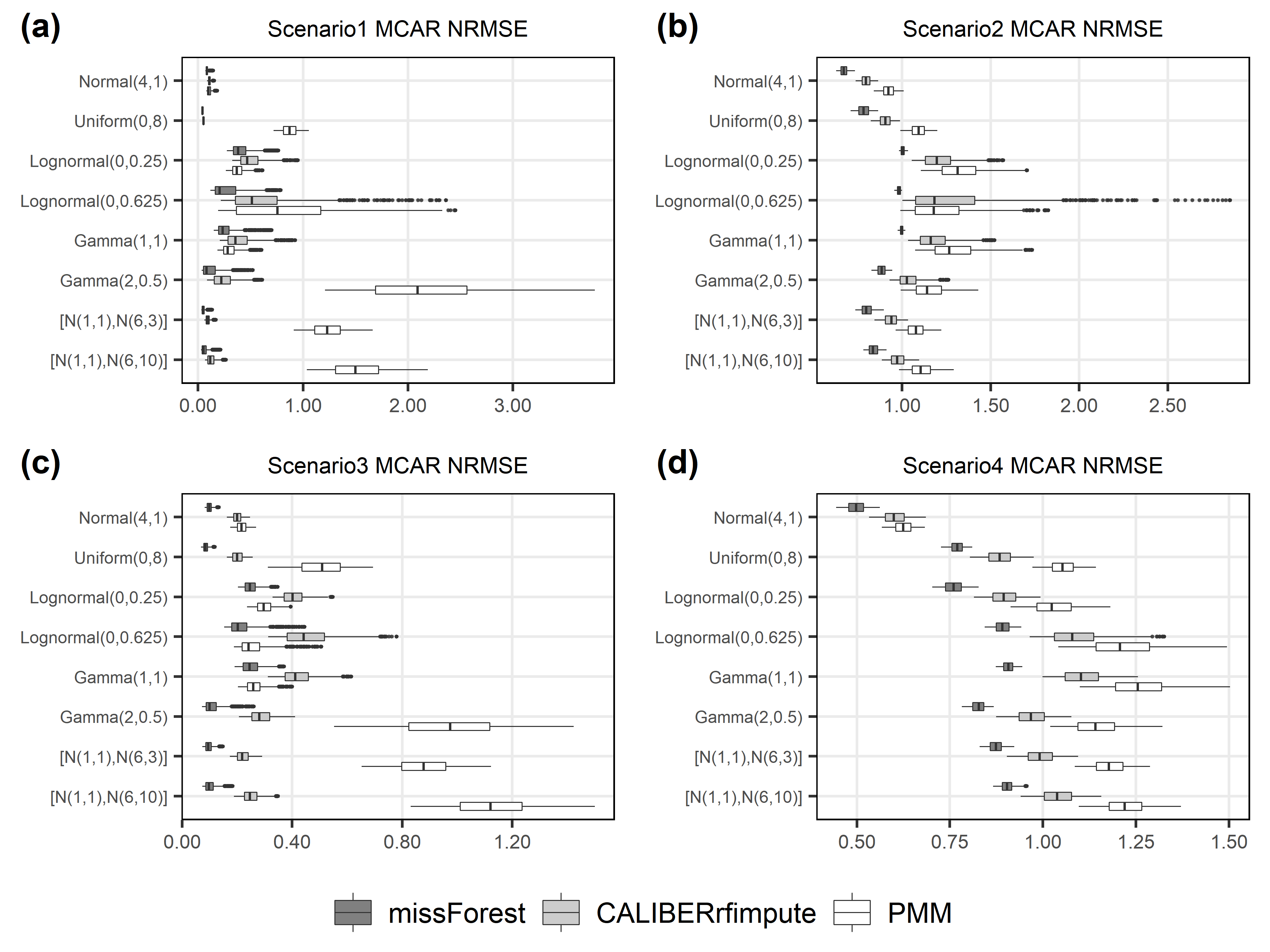

Supplement: Supplementary file 5 — Additional file 5: Figure S4. NRMSE value for MCAR data. [file 12874_2020_1080_MOESM5_ESM.tiff]

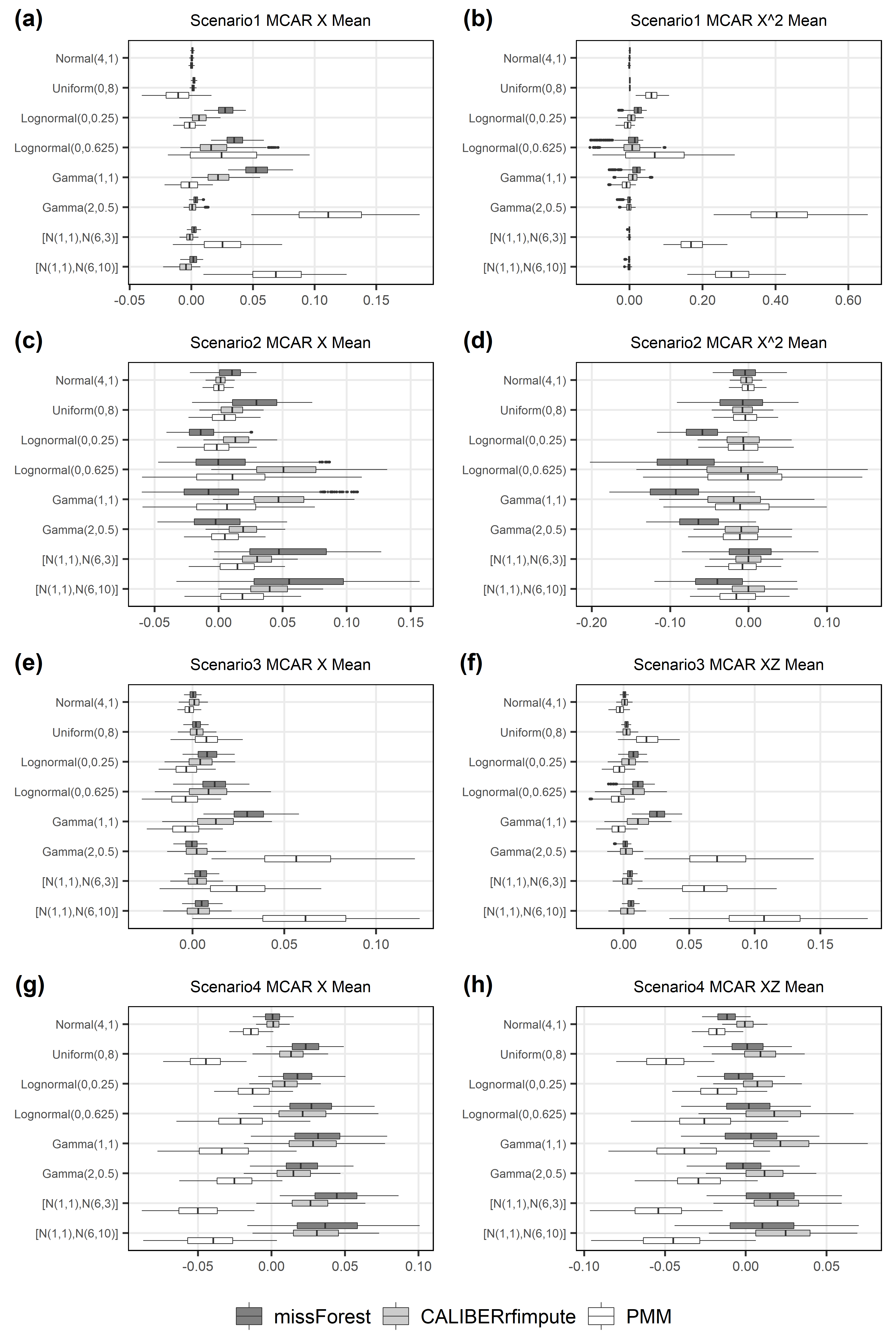

Supplement: Supplementary file 6 — Additional file 6: Figure S5. Relative bias of the estimated mean of imputed variables for MCAR data. [file 12874_2020_1080_MOESM6_ESM.tiff]

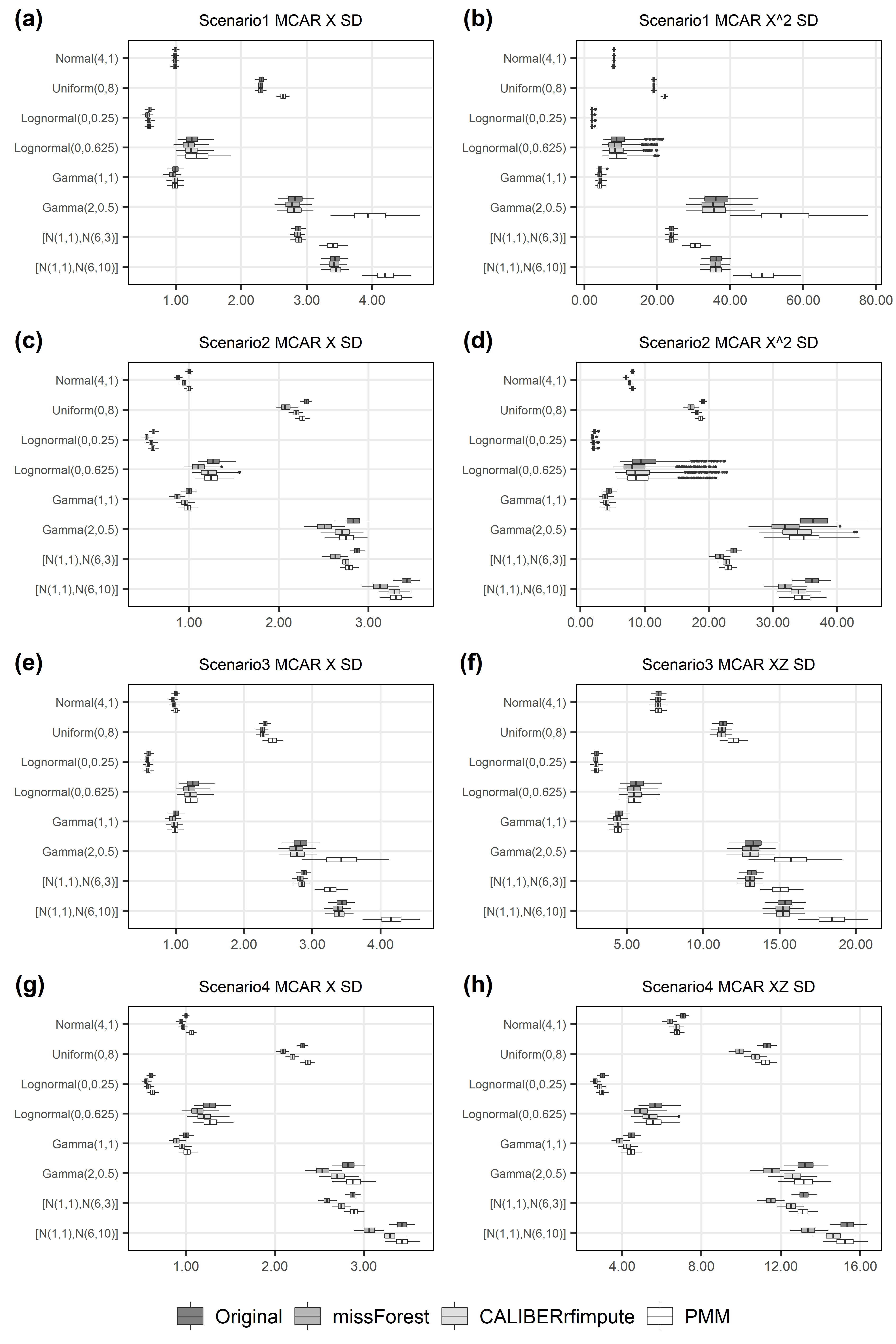

Supplement: Supplementary file 7 — Additional file 7: Figure S6. Standard deviation of imputed variables for MCAR data. [file 12874_2020_1080_MOESM7_ESM.tiff]

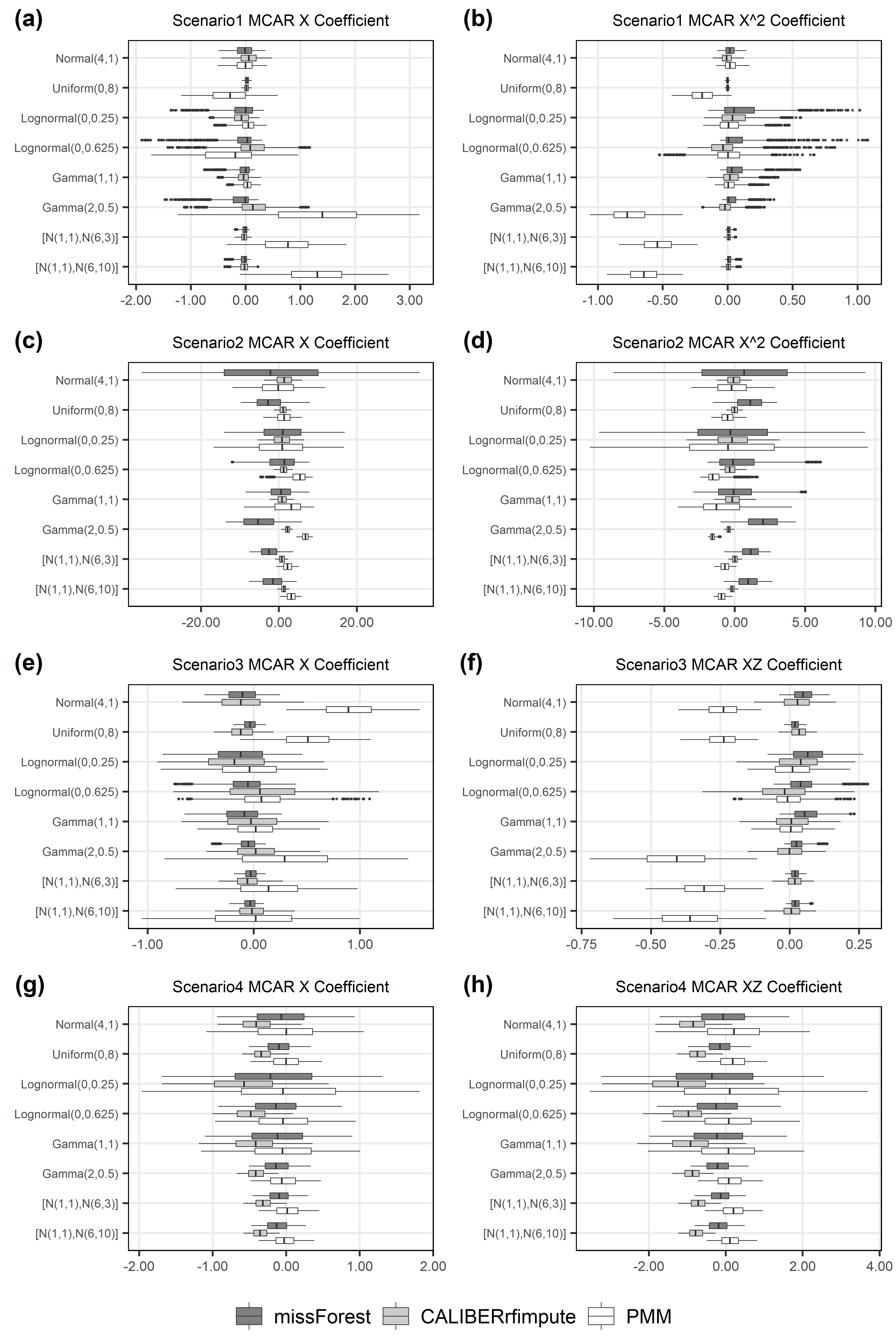

Supplement: Supplementary file 8 — Additional file 8: Figure S7. Relative bias of the estimated regression coefficient of imputed variables for MCAR data. [file 12874_2020_1080_MOESM8_ESM.tiff]

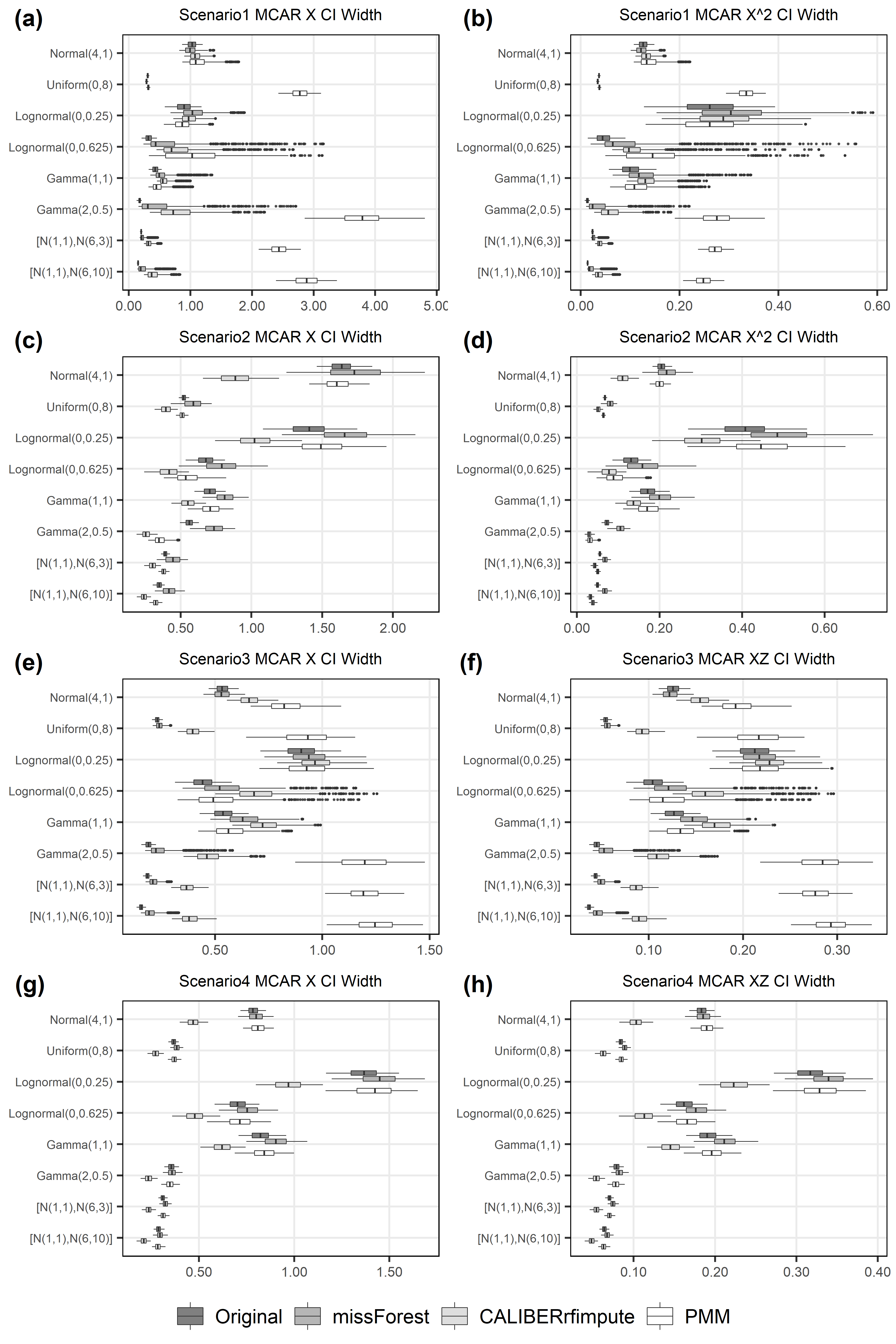

Supplement: Supplementary file 9 — Additional file 9: Figure S8. Width of 95% confidence intervals of the estimated regression coefficients of imputed variables for MCAR data. [file 12874_2020_1080_MOESM9_ESM.tiff]

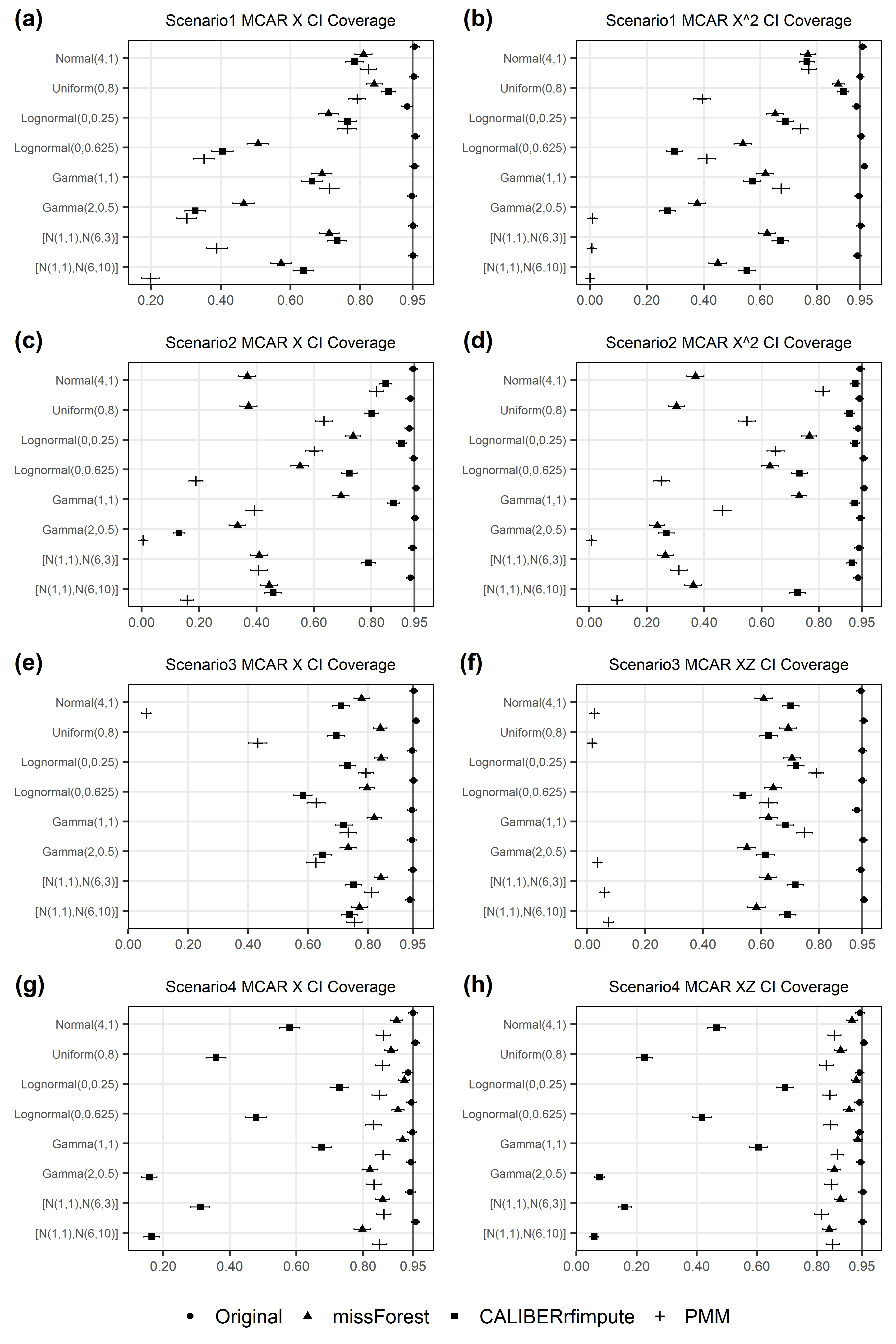

Supplement: Supplementary file 10 — Additional file 10: Figure S9. Coverage of 95% confidence intervals (with binomial proportion confidence intervals) of the estimated regression coefficients of imputed variables for MCAR data. [file 12874_2020_1080_MOESM10_ESM.tiff]

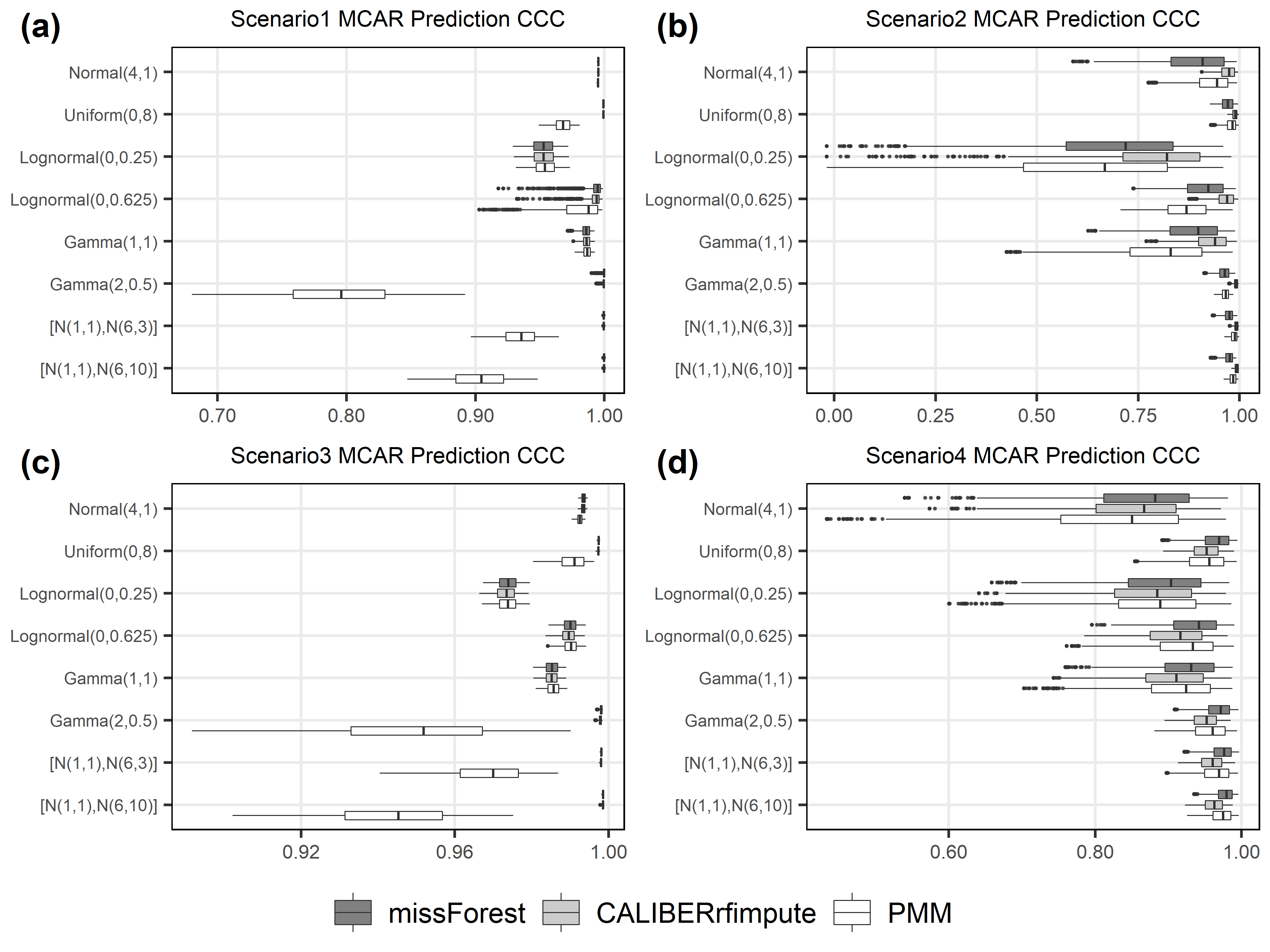

Supplement: Supplementary file 11 — Additional file 11: Figure S10. Lin’s concordance correlation coefficient (CCC) from predictions using models estimated from imputed MCAR data. [file 12874_2020_1080_MOESM11_ESM.tiff]
